# Twitter dataset on public sentiments towards biodiversity policy in Indonesia

**DOI:** 10.1016/j.dib.2023.109890

**Published:** 2023-12-01

**Authors:** Mohammad Teduh Uliniansyah, Indra Budi, Elvira Nurfadhilah, Dian Isnaeni Nurul Afra, Agung Santosa, Andi Djalal Latief, Asril Jarin, Gunarso, Meganingrum Arista Jiwanggi, Nuraisa Novia Hidayati, Radhiyatul Fajri, Ryan Randy Suryono, Siska Pebiana, Siti Shaleha, Tosan Wiar Ramdhani, Tri Sampurno

**Affiliations:** a Research Organization for Electronics and Informatics, National Research and Innovation Agency, Jakarta Pusat 10340, Indonesia; b Faculty of Computer Science, University of Indonesia, Depok 16424, Indonesia; c Universitas Teknokrat Indonesia, Bandar Lampung 35142, Indonesia

**Keywords:** Sentiment analysis, Natural language processing, Indonesian, Health, Environmental management, Food security

## Abstract

In recent years, biodiversity has emerged as a prominent and pressing topic due to the urgent need to address biodiversity loss and the recognition of its connections to climate change and sustainable development. Additionally, increased public awareness and the consideration of economic factors have further underscored the significance of biodiversity conservation. To investigate the sentiment of the Indonesian people towards biodiversity, we conducted a comprehensive data collection on Twitter, focusing on keywords we have set. We amassed a substantial dataset of 500,000 Indonesian tweets from January 2020 to March 2023. These tweets encompassed a wide range of discussions on biodiversity, including its subdomains such as food security, health, and environmental management. Three annotators labeled each tweet with a sentiment class (positive, negative, neutral), or label none for unrelated tweet. The final label was determined using the majority voting method. The tweets with the final label none and those with undecided sentiment class were considered invalid and excluded in the subsequent process. Before labeling, a team of 18 experts jointly developed a labeling guide. This document served as a reference in labeling. After going through a series of processes, including cleaning (removing duplications, irrelevant tweets, and tweets written other than in Indonesian) and preprocessing, we prepared a dataset containing 13,435 tweets. We measured the inter-annotator agreement level, made several models using different algorithms and the K-Fold cross-validation method, and evaluated the models. The Fleiss' Kappa value of the dataset was 0.62187 as the value of the inter-annotator agreement level, and the F1-score value with the best model using the pre-trained IndoBERT model was 0.7959. The Fleiss' Kappa and F1-score values suggest that the annotators have a substantial comprehension and agreement of how to label a tweet, thus ensuring consistency and reliability of our dataset, and the reusability of our dataset is quite suitable for further research on sentiment analysis on biodiversity, respectively. This dataset will benefit various research, including topic modeling, sentiment analysis, public opinion analysis on Twitter, etc., especially biodiversity-related policies.

Specifications Table

**Table:** 

Subject	Biodiversity, Computer Science, Social Science, Natural Language Processing
Specific subject area	Sentiment Analysis on Tweets, Public Opinion Analysis
Data format	Raw Filtered Analyzed
Type of data	Text (CSV-formatted)
Data collection	We gathered a Twitter dataset of around 30 particular biodiversity-related keywords with dates ranging from January 2020 to March 2023. This data was then refined by filtering out irrelevant information, including non-Indonesian language content, non-Biodiversity data, spam, and duplicate entries. Independent analysts undertook the task of manually assigning sentiment labels to the dataset. These eighteen individuals consisted of twelve researchers and engineers specializing in natural language processing, of which two held Ph.D. degrees, nine had MSc degrees, and one had a BSc degree. Additionally, four lecturers and two experts in natural language processing, each with a Ph.D. or MSc degree, contributed to the labeling process. The sentiments were divided into three classes, and the principle of majority voting determined the final class label.
Data source location	Country: Indonesia
Data accessibility	Repository name: Mendeley Data Data identification number: 10.17632/xtk9wsxjjr.4 Direct URL to data: https://data.mendeley.com/datasets/xtk9wsxjjr/4

## 1 Value of the Data

• The dataset was created by collecting information using expert-selected keywords relevant to biodiversity issues widely discussed by the public, covering the subdomains of food security, health, and environmental management. It was meticulously labeled by native speakers through a multi-step process, ensuring consistency and reliability by measuring inter-annotator agreement.
• These data provide valuable insights into public opinion on biodiversity issues on Twitter, serving as a valuable guide for the development of public policies. The dataset validates sentiment and contextual information and enables analysis of public opinion on biodiversity issues, which often encompass diverse viewpoints.
• The government institutions, academics, observers, and communities engaged in sentiment analysis research in the three biodiversity subdomains can utilize this dataset, both for gathering public opinions on biodiversity-related topics and for developing sentiment classification models in the Indonesian language using various artificial intelligence methods.
• The availability of this dataset supports research on sentiment patterns, public perception, and evidence-based decision-making, offering valuable insights into public perspectives on health, food security, environmental management, and biodiversity policies. This dataset can be used to help identify words that tend to convey negative and positive sentiments towards government policies. With this dataset, decision-makers can study how the public reacts to these policies and assess whether these policies receive support or criticism.

## 2 Data Description

We have released a dataset of Twitter's tweets on biodiversity, including issues on its subdomains: food security, health, and environmental management. The tweets’ dates range from January 2020 to March 2023. The tweets originated from user accounts in the Indonesian language. The dataset comprises four files: raw, cleaned-and-selected, sentiment-class labeled, and ready-to-be-trained data.

All dataset is available in the repository Mendeley Data [1].
Data identification number: 10.17632/xtk9wsxjjr.4
Direct URL to data: https://data.mendeley.com/datasets/xtk9wsxjjr/4

a. **Raw data (biodiversity_raw.csv)**

The raw data was collected based on carefully selected keywords, often used on issues currently discussed by the Indonesians. Along with tweet ID, each data in the files has additional information such as keyword and which subdomain the keyword belongs. The raw data comprises 500,000 tweet IDs, keywords, and each tweet's subdomain. These tweets were collected using some keywords listed in Table 1.

b. **Cleaned and selected data (biodiversity_cleaned.csv)**

**Table 1:** List of keywords for each subdomain.

Subdomain	Keywords
Food Security	*ketahanan pangan* (food security)*,* food estate*, swasembada pangan* (food self-sufficiency)*, minyak goreng/migor* (cooking oil)*, lumbung pangan* (food barn)*, sorgum* (sorghum)*, bendungan* (dam)*, waduk/ embung* (reservoir)*, paceklik* (famine)*, diversifikasi pangan* (food diversification)*, kelapa sawit* (palm oil)*, ekstensifikasi pertanian* (agricultural expansion)*, intensifikasi pertanian* (agricultural intensification)*, desa mandiri pangan* (food independent village)*, impor kedelai* (soybean import)*, jewawut/jawawut/ juwawut* (barley)*, gagal panen* (crop failure)*, subsidi pupuk* (fertilizer subsidies)*, impor beras/import beras* (rice imports)
Environmental Management	*kebakaran hutan* (forest fires)*, ekowisata* (ecotourism)*, perubahan iklim* (climate change)*, nikel* (nickel)*, bauksit* (bauxite)*, deforestasi* (deforestation)*, hutan bakau* (mangrove forest)*, daur ulang* (recycle)*, emisi karbon* (carbon emission)*, hutan lindung* (protected forest)*, suaka margasatwa* (wildlife reserve)*, cagar alam* (nature preserve)*, pemanasan global* (global warming)*, keanekaragaman hayati* (biodiversity)*, kendaraan listrik* (electric vehicle), *mobil listrik* (electric car), *motor listrik* (electric motor), *biodiversitas* (biodiversity)
Health	*ketersediaan air bersih* (availability of clean water)*, stunting,* covid/coronavirus/covid-19*, bahan baku obat* (medicinal ingredients)*, obat herbal* (herbal medicine)

The tweets in this dataset were selected by considering the proportion of the number of tweets based on keywords and subdomains. We removed duplicated data, unrelated data to biodiversity, and non-Indonesian data to create a cleaned-and-selected dataset comprising 200,015 tweet IDs, keywords, and the subdomain to which each tweet belongs.

c. **Labeled data (biodiversity_labeled.csv)**

From the cleaned data, we conducted data sampling for the generated labeled dataset as ground truth, called labeled data. This step involved the process of data labeling, which was performed on a data sample comprising 15,323 tweets. The data labeling process was conducted by six distinct groups, with three annotators in each group. Each annotator will assign a sentiment classification (positive, negative, neutral) to each tweet or none for unrelated tweet. The ultimate classification is ascertained by aggregating the majority decision from the three annotators. Consequently, when there is a discrepancy in the labels assigned by each annotator, the tweet is classified as invalid. The degree of agreement among annotators is quantified using the Fleiss' Kappa score.

The Fleiss' Kappa score ranges from -1 to 1, a statistical measure used to assess the level of agreement among annotators when evaluating the same entity [2]. A value close to 1 suggests a high level of understanding that exceeds what would be expected by chance alone. Conversely, a value close to 0 indicates no better agreement than what would be expected by chance. Negative values indicate agreement worse than what would be expected by chance.

To describe the combined Fleiss’ Kappa scores for Food Security, Environmental Management, and Health, we use the term “overall Fleiss’ Kappa score” or “composite Fleiss’ Kappa score.” This term signifies the combined or aggregate measurement of agreement among multiple annotators for the three subdomains of biodiversity: Food Security, Environmental Management, and Health as shown in Table 2.

**Table 2:** Scores of inter-annotator agreement level (labeled dataset).

Subdomain	Fleiss’ Kappa Score
Food Security	0.59130
Environmental Management	0.60425
Health	0.65319
Overall Score	0.62187

**Fig. 1:**
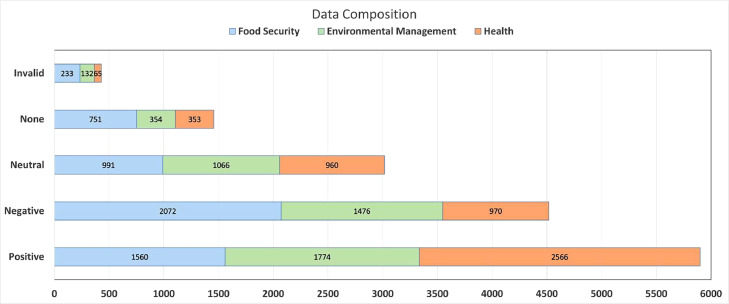
Final Label Distribution of Indonesian Twitter from Three Subdomains.

Fig. 1 illustrates the distribution of positive, negative, and neutral labels and two special labels, none and invalid, across the health, food security, and environmental management subdomains. The health subdomain has the highest count of positive tweets (2566), while food security has the highest count of negative tweets (2,072). Similarly, environmental management has the highest count of neutral tweets (1066), and food security has the highest count of tweets labeled as “none” (751). Regarding invalid tweets, the subdomain health has the lowest count (65). These findings provide valuable insights into the sentiment distribution within each category, contributing to a better understanding of sentiment trends in health, food security, and environmental management discussions on Indonesian Twitter. The labeled dataset contains 15,323 tweet IDs, keywords, subdomains to which the tweet belongs, first annotator label, second annotator label, third annotator label, and final label.

d. **Ready-to-be-trained data (biodiversity_for_modeling.csv)**

For modeling, only tweet data with positive, negative, and neutral final labels were used, so all data labeled with none and invalid were removed. The total tweets obtained were 13,435. This dataset contains tweet IDs, keywords, subdomains to which the tweet belongs, and final label.

**Fig. 2:**
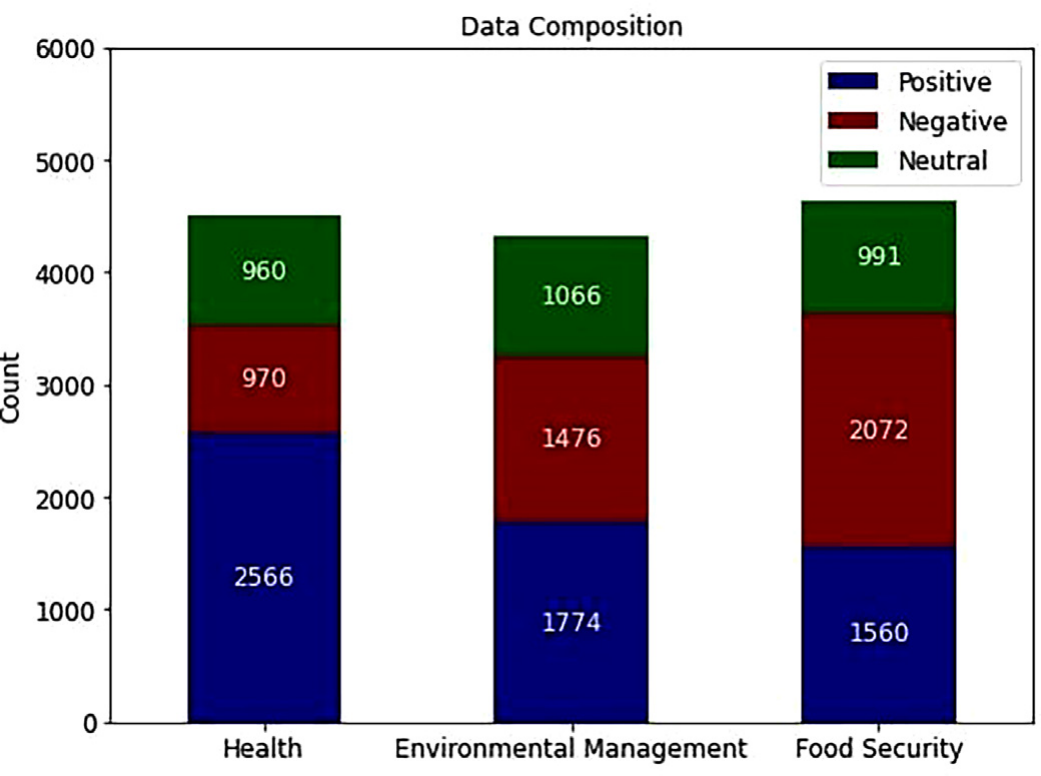
Sentiment-class Distribution in Indonesian Twitter in each Subdomain.

Fig. 2 presents sentiment distribution within the health, food security, and environmental management subdomains in the 4th dataset. It shows a prevalent positive sentiment toward health-related topics, with many positive tweets (2566). However, this category also has significant negative tweets (970) and neutral tweets (960). Similarly, positive sentiments are present in the food security and environmental management subdomains, but there are also notable numbers of negative tweets. Overall, the figure provides an overview of the sentiment composition in the dataset, indicating a mix of positive, negative, and neutral sentiments expressed by Indonesian Twitter users regarding health, food security, and environmental management topics. In Table 3, we utilized word clouds to visualize frequencies of word appearances in each sentiment class within each subdomain, offering a more comprehensive depiction of our data.

**Table 3:** Word Clouds of Sentiment Classes in Each Subdomain.

		Keywords		
		Positive	Negative	Neutral
Subdomain	Health			
	Food Security			
	Environmental Management			

The figures in Table 3 depict visual representations of frequently used words within health, food security, and environmental management subdomains, categorized according to their respective positive, negative, and neutral sentiment classes. Within the realm of health, the term “*cegah*” (meaning “prevent” in English) holds a prominent position within the positive sentiment category. This phenomenon can be linked to various government and community initiatives, public guidance and perspectives, and diverse news coverage, all aimed at collectively mitigating the spread of stunting and its associated consequences, such as hindering societal progress. In the negative sentiment class, the words “*angka*” (numbers), “*gizi*” (nutrition), and “*balita*” (toddlers) exhibit a notable frequency of occurrence. Shifting our focus to the realm of food security, it is evident that three key terms, specifically “*pangan*” (food), “*masyarakat*” (society), and “*pertanian*” (agriculture), prominently represent the positive sentiment category. The term that exhibits the highest frequency within the negative sentiment category is “*harga*,” which translates to “price” in English. Moreover, within environmental management, the term “PLN” (referring to a government-owned electricity company) exhibits a prevailing presence in the positive sentiment category. In contrast, the negative sentiment category is predominantly characterized by “*banjir*” (denoting a flood event).

## 3 Experimental Design, Materials and Methods

**Fig. 3:**
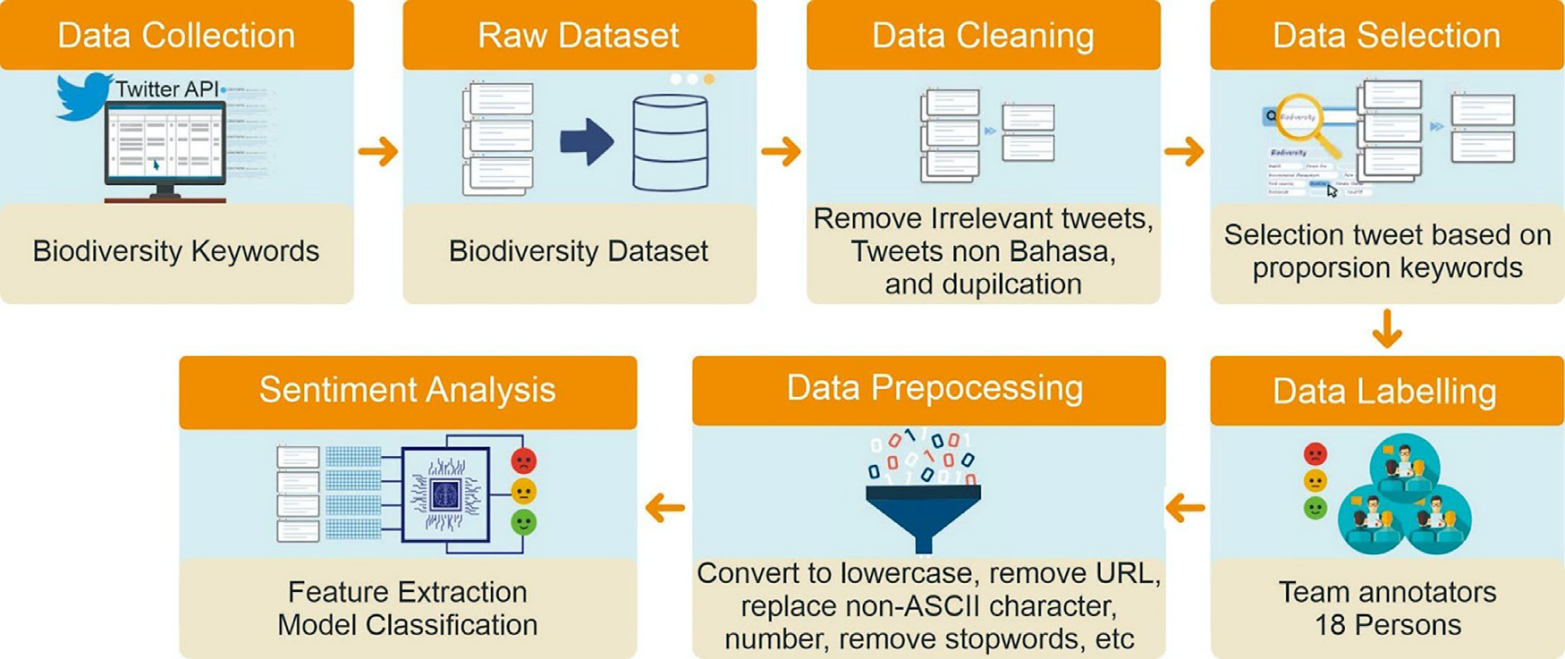
Process flow of datasets creation and evaluation.

Fig. 3 shows the process flow of creating and evaluating our dataset. The explanation of each process is described below.

a. **Raw Data Collection**

Before collecting Twitter data, we decided on several keywords in each subdomain as the phrase to be searched among Twitter's tweets. The keywords were chosen since they were assumed as part of viral issues in the Indonesian communities. Hence, it was hoped that many tweets would contain those keywords. Twitter data was collected using the Twitter API, and the collected data dates ranged from January 2020 to March 2023. The total amount of the collected data was 500,000.

b. **Data Cleaning**

We found a lot of data in the raw dataset that could not be used, such as duplicated data, unrelated data to biodiversity, including the three subdomains, and data not written in Indonesian. We then removed those data from the raw dataset, which produced 200,015 data.

c. **Data Selection**

After cleaning the data, we picked the data according to the proportion of collected tweets. The total number of chosen tweets was 15,323 for all three subdomains. Data selection was carried out by applying data sampling with the parameters of a 99% confidence level and a 1% confidence interval. Each subdomain had approximately 5,000 tweets, and each subdomain received an equal proportion in this dataset to ensure that it can effectively represent the biodiversity topics discussed.

d. **Data Labeling**

Before labeling each tweet in the raw data, a team of annotators comprised of 18 persons created a labeling guidance document [3]. The document included detailed instructions on annotating a tweet with a sentiment class label (positive, negative, neutral) or “none” label and some examples. In this labeling phase, a “none” label was introduced to categorize tweets that are unrelated to biodiversity. This was necessary because during the preceding data cleaning stage, many tweets were not filtered out due to the presence of keywords, even though they were not related to the biodiversity topic. These tweets included advertisements, recipes, excerpts from short stories/novels/dialogues, but they contained search keywords either in their content or in the hashtags used. Hence, human annotation was required to mark them with the “none” label. Generally, health, environmental management, and food security issues on Twitter are of a general nature, and in practice, they can be understood by every labeler, enabling them to provide reasonable judgments.

Referring to the labeling guidance document, each annotator labeled a set of 800 tweets from each subdomain functioning as a sample set. Three annotators labeled a tweet. The results were then verified by the majority voting method, and the level of inter-annotator agreements was measured. The labeling guidance document was revised according to issues found during the verification process. These steps were repeated until all problems were resolved, and the labeling guidance document was considered ready to be used as a reference in the labeling process. In the labeling guidelines we created, the labeling of tweets as news is considered based on references from several previous studies [4], [5], [6]. It has been indicated by these studies that news is not only served to present factual information but also used as a means to convey various emotional states, including sentiments like empathy, joy, apprehension, rage, and more. The importance of paying attention to headlines has been emphasized by these studies because news typically contains a high emotional content, as major national or international events are described, and they are written in a style intended to capture readers' attention. It has been suggested by these studies that these headlines potentially determine how many people read the news and even that headlines can influence the way users comprehend the entire related content and shape their perspective.

Table 4 below shows the characteristics of each sentiment class label defined in the labeling guidance document.

a. **Data Preprocessing**

**Table 4:** Characteristics of sentiment class label as defined in the labeling guide document.

Sentiment class label	Characteristics	Examples (with English translation)
Positive	• Containing news with a positive connotation. • Containing words of invitation such as '*ayo* (lets),' '*mari* (lets),' etc., on positive things that illustrate the optimistic spirit of the tweet's content on a biodiversity issue. • Containing implementation words such as *'bahas* (to elaborate),' *'diskusi* (to discuss),' *'selesaikan* (to finish),' *'menghadiri rapat* (to join a meeting),' etc., for positive things things illustrating there have been efforts to address a biodiversity issue. • Containing positive suggestions • Containing positive expectations	“*Pekanbaru - Kepala Dinas Pengendalian Penduduk Keluarga Berencana Pemberdayaan Perempuan dan Perlindungan Anak Hadiri rapat Koordinasi Percepatan penurunan Stunting. Lintas sektor dan lintas program Tingkat Provinsi…*” (“Pekanbaru - Head of Family Planning Population Control Service for Women's Empowerment and Child Protection Attends a Coordination meeting for Accelerating Stunting Reduction. Cross-sectoral and cross-program Provincial level…”) “*Buku Inspirasi Resep Megawati Soekarnoputri berikan pedoman praktis atasi stunting*” (“The Inspirational Recipe Book by Megawati Soekarnoputri provides practical guidelines to overcome stunting.")
Negative	• Containing news with a negative connotation. • Containing words of invitation such as '*ayo* (lets),' *'mari* (lets),' etc., on negative things that illustrate the pessimistic spirit of the tweet's content on a biodiversity issue. • Containing implementation words such as *'bahas* (to elaborate),' *'diskusi* (to discuss),' *'selesaikan* (to finish),' *'menghadiri rapat* (to join a meeting),' etc., for negative things illustrating there have been efforts to address a biodiversity issue. • Containing negative suggestions • Containing negative expectations	“*Prevalensi stunting di NTT 43,82%, tertinggi dari 34 propinsi*.” (“The prevalence of stunting in NTT is 43.82%, the highest among 34 provinces.”) *“Batalkan Impor Beras, Serap Gabah Petani 100 Persen !*” (“Cancel Rice Imports, Absorb Grain Farmers 100 Percent!”)
Neutral	• Generic tweet stating a definition • Containing informative facts but no positive or negative sentiments • Discussing a government policy, there is no indication of a particular feeling or emotion that can indicate the tweet's sentiment. • Containing comparisons	“*Hal ini tidak lepas dari cetak biru BRIvolution 2.0 yang diterapkan sejak awal pandemi covid 19*” (“This cannot be separated from the BRIvolution 2.0 blueprint implemented since the beginning of the covid 19 pandemic.”) “*Direktur Informasi dan Komunikasi Pembangunan Manusia dan Kebudayaan, Dirjen Informasi dan Komunikasi Publik Kemenkominfo Wiryanta mengatakan, bonus demografi menjadi perhatian utama pemerintah*.” (“Director of Information and Communication for Human Development and Culture, Director General of Information and Public Communication of the Ministry of Communication and Informatics Wiryanta said that the demographic bonus is the government's primary concern.”)
None	• Not related to biodiversity, such as containing product advertisements, food recipes, etc. • Containing non-Indonesian words • Containing absurd jokes or joking terms	“*Aku lagi nyoba jualan skincare & obat" herbal, udah lama sih jadi member, tapi gak diseriusin soalnya masih sibuk ditoko alat tulis sekolah, sekarang udah gak ada yg kesekolah baru deh kepikiran buat serius*” (“I'm trying to sell skincare & herbal medicine, I've been a member for a long time, but I didn't take it seriously because I'm still busy at the school stationery shop, and now no one's going to school. I'm thinking about getting serious.”) “*06 Jan 2023 Status Terkini COVID-19 Malaysia Maklumat lanjut sila layari*” (“06 Jan 2023 Current Status of COVID-19 Malaysia For further information, please review”)

We conducted a series of preprocessing phases [7] to generate a dataset (biodiversity_for_modelling.csv) for sentiment classification modeling. The initial stage encompassed converting all text to lowercase to ensure uniformity and eliminate potential stemming discrepancies from capitalization. Subsequently, the data underwent URL removal, and punctuation was substituted with spaces, except for apostrophes, while non-ASCII characters were replaced with their nearest ASCII equivalents. After that, we normalized slang and typos, standardized word variations, and mitigated the influence of slang and typographical errors. Then, numerical values and non-ASCII characters were excluded, and instances of multiple consecutive whitespaces were condensed to a single whitespace. Finally, stopword removal was implemented using the Sastrawi library [8], except for adverbial words, which were retained in the stop words list due to their inherent significance and influence on sentence sentiment. These preprocessing steps resulted in the ready-to-be-trained dataset with a total of 13,435 data.

a. **Sentiment Analysis Model Creation and Evaluation**

We used two scenarios for creating sentiment analysis models. In the first scenario, we utilized the IndoBERT Tweet pre-trained model [9]. Two fully connected layers then used the sentence embeddings created from IndoBERT Tweet pre-trained model as input to generate the sentences sentiment class. We used three different configurations of activation function for the first connected layer: non-activation, Gaussian Error Linear Unit (GELU), and norm hyperbolic tangent (norm tanh). Among these, the configuration with the GELU activation function showed promising outcomes, achieving a best accuracy of 0.8263 and a best F1-score of 0.7959. We also experimented using preprocessed text dataset using the same setup and obtained accuracies ranging from 0.7765 to 0.7914, and F1-score varied from 0.7441 to 0.754.

In the second scenario, we used the Term Frequency – Inverse Document Frequency (TF-IDF) method to extract features of the labeled dataset, which were then used in various traditional machine learning algorithms such as Logistic Regression [10], Support Vector Classifier (SVC) [11], Light Gradient Boosting Machine (LGBM) [12], Random Forest [13], Extreme Gradient Boosting (XGB) [14], AdaBoost [15], and Decision Tree [16]. The Logistic Regression and SVC classifiers achieved the highest accuracies of 0.72115 and 0.71967, respectively, while LGBMClassifier, Random Forest Classifier, and XGB Classifier exhibited slightly lower accuracies. However, it is crucial to consider both accuracy and F1-score, which assumes precision and recall. The Logistic Regression and SVC classifiers also achieved the highest F1-score of 0.70619 and 0.70230, respectively.

Table 5 summarizes the performance of all classifiers, and the best model is the one created using IndoBERT Tweet pre-trained model with the GELU activation method. These results provide valuable insights into the most effective combinations of word embeddings and classifiers for sentiment analysis in biodiversity, offering guidance for future research in this domain.

**Table 5:** Classification performance of ten classifiers using 10-fold cross-validation.

Data type	Classifier	Text Representation	Best Accuracy	Average Accuracy	Best F1-Score	Average F1-Score
Raw text	IndoBERT (non-activation)	Word embedding (Internal BERT)	0.8256	0.788	0.7941	0.756
	IndoBERT (GELU)	Word embedding (Internal BERT)	0.8263	0.793	0.7959	0.760
	IndoBERT (norm tanh)	Word embedding (Internal BERT)	0.8182	0.788	0.7975	0.754
Preprocessed text	IndoBERT (GELU)	Word embedding (Internal BERT)	0.7765	0.760	0.7441	0.722
	IndoBERT (norm tanh)	Word embedding (Internal BERT)	0.7914	0.759	0.754	0.721
	Logistic Regression	TF-IDF	0.72115	0.69485	0.70619	0.68164
	SVC	TF-IDF	0.71967	0.69404	0.70230	0.67671
	LGBMClassifier	TF-IDF	0.70266	0.6695	0.6933	0.66205
	Random Forest Classifier	TF-IDF	0.70266	0.66707	0.69116	0.65548
	XGB Classifier	TF-IDF	0.68491	0.65734	0.67403	0.64722
	AdaBoost Classifier	TF-IDF	0.622	0.604	0.618	0.585
	DecisionTree Classifier	TF-IDF	0.60429	0.56831	0.60389	0.56816

## Limitations

The dataset that has been published is limited to Indonesian Twitter data with a theme of biodiversity, encompassing health, environmental management, and food security issues. Therefore, preliminary testing is deemed necessary to apply our dataset to Indonesian Twitter data with different themes.

## Ethics Statement

The gathered information was acquired and shared in accordance with Twitter's developer policy for 2023 [17]. The Twitter data was handled responsibly, with consideration for the privacy and control of its users. In the analysis, measures were taken to safeguard the privacy of Twitter users and maintain their anonymity without divulging specific details.

## CRediT authorship contribution statement

**Mohammad Teduh Uliniansyah:** Project administration, Data curation, Methodology, Writing – review & editing. **Indra Budi:** Supervision, Methodology, Writing – review & editing. **Elvira Nurfadhilah:** Data curation, Methodology, Visualization, Investigation, Writing – original draft, Writing – review & editing. **Dian Isnaeni Nurul Afra:** Data curation, Methodology, Writing – original draft, Writing – review & editing. **Agung Santosa:** Methodology, Writing – review & editing. **Andi Djalal Latief:** Writing – review & editing. **Asril Jarin:** Writing – review & editing. **:** Writing – review & editing. **Meganingrum Arista Jiwanggi:** Writing – review & editing. **Nuraisa Novia Hidayati:** Methodology, Visualization, Writing – original draft, Writing – review & editing. **Radhiyatul Fajri:** Data curation, Visualization, Writing – review & editing. **Ryan Randy Suryono:** Writing – review & editing. **Siska Pebiana:** Methodology, Writing – review & editing. **Siti Shaleha:** Data curation, Writing – review & editing. **Tosan Wiar Ramdhani:** Writing – review & editing. **Tri Sampurno:** Writing – review & editing.

## Data Availability

Indonesian Biodiversity-related Tweets Including Health, Food Security, and Environmental Management Issues for Sentiment Analysis (Original data) (Mendeley Data)

## References

[1] Uliniansyah M.T., Santosa A., Latief A.D., Jarin A., Afra D.I.N., Nurfadhilah E., Gunarso G., Budi I., Hidayati N.N., Fajri R., Suryono R.R., Pebiana S., Shaleha S., Ramdhani T.W., Sampurno T., Jiwanggi M.A., Raif M.I., Nanda T. (2023). Indonesian biodiversity-related tweets including health, food security, and environmental management issues for sentiment analysis. Mendeley Data.

[2] Fleiss J.L. (1971). Measuring nominal scale agreement among many raters. Psychol. Bull..

[3] Latief A.D., Jarin A., Uliniansyah M.T., Nurfadhilah E., Afra D.I.N. (2023). Proceedings of the International Conference on Computer, Control, Informatics and Its Applications (IC3INA).

[4] Lei J., Rao Y., Li Q., Quan X., Wenyin L. (2014). Towards building a social emotion detection system for online news. Futur. Gener. Comput. Syst..

[5] Reis J., Benevenuto F., de Melo P.O.S.V., Prates R., Kwak H., An J. (2015).

[6] Strapparava C., Mihalcea R. (2007). Proceedings of the 4th International Workshop on Semantic Evaluations.

[7] Pebiana S., Hidayati N.N., Afra D.I.N., Nurfadhilah E., Prafitia H.A., Prihantoro J., Fajri R., Uliniansyah M.T., Santosa A., Aini L.R., Sahreza Y., Subekti A.H.K.M., Pinem J.G., Alfin M.R., Septadi A., Shaleha S., Wibowanto G.S., Jarin A., Gunarso A.D.L., Riza H. (2022). Proceedings of the 25th Conference of the Oriental COCOSDA International Committee for the Co-Ordination and Standardisation of Speech Databases and Assessment Techniques (O-COCOSDA).

[8] Sastrawi, PyPI, Ht t ps://Pypi.Org/Project/Sastrawi/. (2016). https://pypi.org/project/Sastrawi/ accessed July 29, 2023).

[9] Koto F., Rahimi A., Lau J.H., Baldwin T. (2020). Proceedings of the 28th International Conference on Computational Linguistics, International Committee on Computational Linguistics.

[10] McCullagh P., Nelder J.A. (1989).

[11] Cortes C., Vapnik V. (1995). Support-vector networks. Mach. Learn..

[12] Ke G., Meng Q., Finley T., Wang T., Chen W., Ma W., Ye Q., Liu T.Y. (2017). Proceedings of the 31st International Conference on Neural Information Processing Systems.

[13] Ho T.K. (1995). Proceedings of 3rd International Conference on Document Analysis and Recognition.

[14] Chen T., Guestrin C. (2016). Proceedings of the 22nd ACM SIGKDD International Conference on Knowledge Discovery and Data Mining.

[15] Schapire R.E. (2013). Empirical Inference.

[16] Wu X., Kumar V., Quinlan J.R., Ghosh J., Yang Q., Motoda H., McLachlan G.J., Ng A., Liu B., Yu P.S., Zhou Z.H., Steinbach M., Hand D.J., Steinberg D. (2008). Top 10 algorithms in data mining. Knowl. Inf. Syst..

[17] Twitter API, Twitter Inc., Twitter Developer Policy, (2023). https://developer.twitter.com/en/developer-terms/policy (accessed July 29, 2023).

